# Readmissions in Inflammatory Bowel Diseases: A Five-Year Retrospective Study in a Romanian Emergency County Hospital

**DOI:** 10.7759/cureus.97106

**Published:** 2025-11-17

**Authors:** Paul Grama, Vladimir Zadea, Naomi-Adina Ciurea, Simona M Bataga

**Affiliations:** 1 1st Internal Medicine Department, George Emil Palade University of Medicine, Pharmacy, Science and Technology of Târgu Mureș, Târgu Mureș, ROU; 2 Department of Gastroenterology, Târgu Mureș Emergency Clinical County Hospital, Târgu Mureș, ROU; 3 Doctoral School, George Emil Palade University of Medicine, Pharmacy, Science and Technology of Târgu Mureș, Târgu Mureș, ROU; 4 Department of Internal Medicine, Târgu Mureș Emergency Clinical County Hospital, Târgu Mureș, ROU

**Keywords:** complications, crohn’s disease, hospital readmission, inflammatory bowel disease, risk factors, ulcerative colitis

## Abstract

Background: Hospital readmissions represent a significant problem in the management of chronic diseases, provoking a substantial burden both on patients and the national healthcare system. Chronic diseases such as chronic obstructive pulmonary disease, diabetes mellitus, heart failure, and liver cirrhosis have documented risk factors, and guidelines are being continuously developed for reducing readmissions. Despite advancements, readmission patterns in some chronic conditions still remain underexplored. Recurrent hospitalizations among inflammatory bowel disease (IBD) patients may reflect disease severity, frequency of complications, and inadequate outpatient management, which can negatively impact quality of life and increase financial strain on healthcare systems. This study aimed to characterize readmission frequency, timing, and causes in Crohn’s disease (CD) vs ulcerative colitis (UC) patients over a five-year period, and to identify factors associated with readmissions.

Methods: We conducted a retrospective cohort study at the Târgu Mureș Emergency Clinical County Hospital using electronic records from 2018 to 2023. Adult patients discharged with a diagnosis of CD or UC were identified by International Classification of Diseases, 10th Revision (ICD-10) coding. Data collected included IBD subtype, number of admissions per patient, time intervals between admissions, presenting symptoms, admitting department, and in-hospital complications. Descriptive statistics were performed to compare CD and UC outcomes.

Results: A total of 79 IBD patients accounted for 198 hospital admissions. Of these, 32 (41%) patients had CD and 47 (59%) had UC. CD patients experienced significantly more frequent readmissions, with a shorter mean interval between admissions (172 days for CD vs 487 days for UC, p<0.01). Length of stay per admission was similar between groups (p=0.18). The most common presentation reasons that led to admission in CD were abdominal pain (52 cases, 55%), rectorrhagia (35 cases, 37%), and fever (35 cases, 37%), whereas in UC they were abdominal pain (65 cases, 63%), diarrhea (48 cases, 46%), and rectorrhagia (27 cases, 26%). The majority of admissions in both groups were to the Gastroenterology department (CD: 41 cases (44%), UC: 58 cases (56%)) or surgery (CD: 35 cases (37%), UC: 31 cases (30%)), with a minority to Internal Medicine (CD: 4 cases (4%), UC: 7 cases (7%)) or other services. Key complications differed: CD patients had high rates of perianal abscesses (6 cases, 18%) and fistulas (1 case, 4%), while UC patients more frequently developed intestinal obstruction/occlusion (8 cases, 17%) or hemorrhoids (3 cases, 6%).

Conclusion: IBD readmissions are common, with CD patients prone to earlier and more frequent hospital returns than UC patients, likely due to more frequent disease relapses and complications. Both groups often present with severe symptoms (pain, bleeding) necessitating acute care. Our findings underscore the need for proactive strategies, including optimizing outpatient management and addressing modifiable risk factors such as psychiatric comorbidities, corticosteroid use, and *Clostridioides difficile* infection, to reduce preventable readmissions.

## Introduction

Hospital readmission is recognized as a key quality metric in chronic diseases, reflecting both the severity of illness and the effectiveness of outpatient management. In conditions such as heart failure, chronic lung disease, and diabetes, readmission rates have been closely studied, and targeted interventions have reduced preventable rehospitalizations. However, for inflammatory bowel disease (IBD), comprising Crohn’s disease (CD) and ulcerative colitis (UC), the contributors to hospital readmission are less thoroughly characterized. IBD affects millions worldwide and often entails recurrent flares requiring hospital care [1]. Frequent admissions not only impose physical and emotional burdens on patients but also drive up healthcare costs.

Previous studies indicate that a substantial minority of IBD patients experience early readmission after hospital discharge. Reported 30-day readmission rates range from about 7% to 14%, and up to 20%-25% of IBD admissions are followed by readmission within 90 days. Moreover, CD and UC might have different readmission patterns; national data suggest CD patients have slightly higher odds of readmission than UC patients, potentially reflecting CD’s propensity for complications such as strictures and fistulae. Nonetheless, disease activity alone does not fully explain readmissions [2,3]. Recent research has shifted attention toward modifiable risk factors and comorbidities. In particular, psychiatric disorders (such as anxiety and depression) and chronic pain syndromes have emerged as significant predictors of IBD readmission. Barnes et al. found that in a nationwide cohort, anxiety and depression were associated with ~30% increased odds of 90-day readmission in both CD and UC [1]. Similarly, an Iranian multicentric study reported that chronic corticosteroid use at discharge was linked to a >4-fold higher risk of 90-day readmission, and chronic pain to an even higher risk, highlighting the impact of poorly controlled symptoms on recurrent hospitalization [3].

*Clostridioides difficile* infection (CDI) is another factor compounding IBD admissions. IBD patients are more susceptible to CDI, and concurrent CDI can masquerade as or trigger IBD flares. CDI is associated with worse outcomes in IBD, including prolonged hospital stay, urgent colectomy, and higher mortality. Notably, one study found that UC patients undergoing colectomy during a CDI had significantly higher 30-day readmission rates. Corticosteroid therapy, while often necessary for acute IBD flares, may increase the risk of CDI development and has been linked to greater long-term healthcare utilization in IBD. These findings show that factors beyond intrinsic disease severity, including infections and medication effects, contribute to readmission risk [4].

Against this background, we conducted a single-center retrospective study to evaluate the patterns of hospital readmissions in CD and UC patients over five years at a Romanian tertiary hospital. We specifically aimed to compare readmission frequencies between CD and UC, delineate the typical reasons for readmission and in-hospital complications, and relate our findings to known risk factors such as psychiatric comorbidities, steroid use, and CDI. By identifying the characteristics of patients with frequent readmissions, we aim to inform targeted strategies to keep IBD patients out of the hospital whenever possible.

The present study aimed to characterize and compare the frequency, timing, and causes of hospital readmissions among patients with CD and UC over a five-year period in a Romanian tertiary center, contributing regional evidence to the broader international understanding of IBD management.

## Materials and methods

Study design and setting

We performed a retrospective observational study at the Târgu Mureș Emergency Clinical County Hospital, a tertiary academic center in Romania. The study period spanned five years, from January 2018 to December 2023. This hospital is a referral center for gastroenterology, serving a mixed urban and rural population. Ethical approval was obtained from the hospital review board (waiver of informed consent due to the retrospective design and use of de-identified data). Although the study population was limited to 79 patients from a single tertiary center, the hospital serves as a primary referral facility for IBD in the region, ensuring consistent case capture and standardized diagnostic coding.

Patient identification 

Using the hospital’s electronic medical record and discharge database, we identified all patients discharged during 2018-2023 with a diagnosis of CD or UC. International Classification of Diseases, 10th Revision (ICD-10) coding within the diagnosis-related group (DRG) system was used to capture IBD diagnoses: codes K50.x for CD and K51.x for UC. Both primary and secondary discharge diagnoses were queried to ensure all hospitalizations related to IBD or its complications were included. We included adult patients (age ≥18 years) with confirmed IBD diagnoses. Patients with indeterminate colitis were excluded. If a patient had multiple admissions, all their admissions in the study period were captured. For patients transferred from other facilities, only the admissions to our hospital were counted.

We defined a readmission as any unplanned hospital admission occurring after initial discharge, for either IBD relapse or related complications. To focus on patterns of recurrence, we excluded from analysis any patient who only had a single admission in the period (i.e., no readmission). In the final cohort, each patient had at least two admissions (an index admission and at least one readmission).

Data collection

From the medical records, we abstracted the following variables for each patient: IBD subtype (CD or UC) and the total number of hospital admissions during 2018-2023. For each admission, we recorded the date of admission and discharge, the department or service of hospitalization, the primary reason for presentation (chief complaint or precipitating event), and any key in-hospital interventions or complications. We categorized presentation reasons as gastrointestinal symptoms (e.g., abdominal pain, diarrhea, hematochezia/rectal bleeding, and vomiting), systemic symptoms (e.g., fever), or others (e.g., extraintestinal manifestations). Admission departments were grouped into Gastroenterology, General Surgery, Internal Medicine, and “Other” (including specialties such as Orthopaedics or Gynaecology, if applicable). Major IBD-related complications during hospitalization were noted, such as perianal abscesses, fistulas, intestinal obstruction (defined as bowel obstruction on imaging or requiring surgical intervention), severe bleeding, need for urgent surgery, or new diagnoses (e.g., colorectal neoplasia). We also recorded if CDI was confirmed during any admission (positive stool toxin/PCR test).

For each patient, we calculated the interval between admissions as an indicator of readmission frequency. This was defined as the number of days between consecutive hospital discharges and admissions for that patient, averaged over the patient’s sequence of admissions. We also noted the length of stay (LOS) for each admission (days from admission to discharge) and compared the average LOS between the CD and UC groups.

Statistical analysis 

Data management was performed in Microsoft Excel (Microsoft Corp., Redmond, WA, US), and analyses were carried out in IBM SPSS Statistics (IBM Corp., Armonk, NY). Normality of quantitative variables was assessed using the Kolmogorov-Smirnov test. Quantitative data are reported as mean (or median where appropriate). Between-group comparisons of quantitative variables used the independent-samples Student’s t test. Categorical variables are presented as n (%) and were compared using Fisher’s exact test. All tests were two-sided, and p < 0.05 was considered statistically significant. Outcomes were analyzed both at the admission level and at the patient level; the denominator used is indicated for each result. Missing data were handled by complete-case analysis.

## Results

Cohort characteristics and admission frequency 

**Figure 1:**
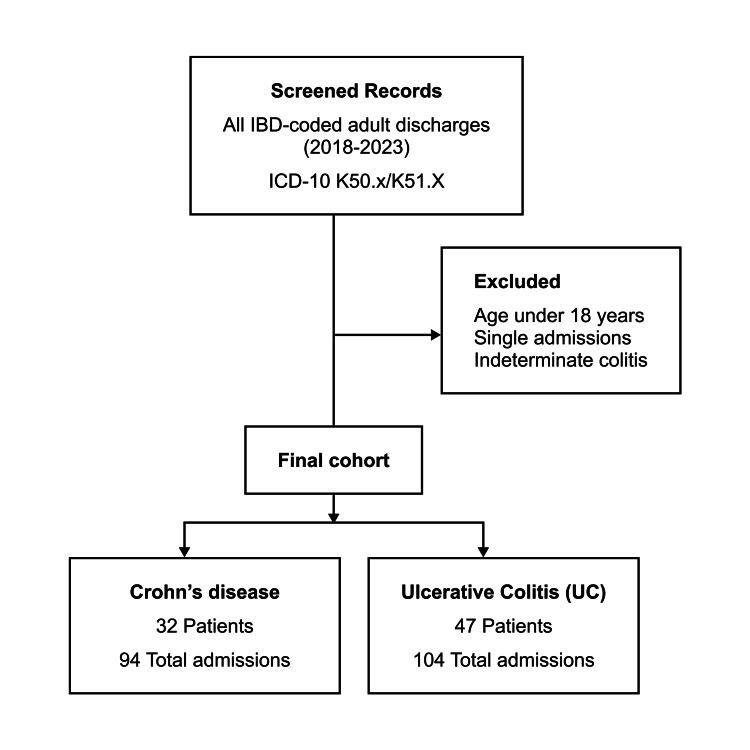
Flow diagram of patient selection and diagnosis breakdown Screening and exclusions for the study cohort (2018-2023, ICD-10 K50.x/K51.x), yielding 79 adults with IBD: CD n = 32 (94 admissions) and UC n = 47 (104 admissions); exclusions included <18 years, single admissions, and indeterminate colitis

Seventy-nine patients met the inclusion criteria, contributing a total of 198 IBD-related hospital admissions over the five-year period. Among these patients, 32 (41%) had CD and 47 (59%) had UC. The median number of admissions per patient was two (range: two to seven). Figure 1 presents a flow diagram of patient selection and diagnosis breakdown in the study.

Time between readmissions

We found that CD patients were readmitted significantly sooner on average than UC patients. The mean interval between hospital admissions was 172 days for CD (approximately 5.7 months) vs 487 days for UC (approximately 16.2 months), a difference that was highly significant (p<0.01). In practical terms, a typical CD patient had about threefold more frequent hospitalizations than a typical UC patient over the five years. The shorter readmission interval in CD remained evident even when excluding a few outliers with multiple recurrent admissions. In contrast, many UC patients, after their index admission, had only one subsequent readmission or long relapse-free periods.

LOS

The duration of each hospitalization was similar between groups. CD admissions had a mean LOS of 8.4 days (±4.1) vs 9.6 days (±5.2) for UC, and this difference was not statistically significant (p=0.18). Thus, while Crohn’s patients returned to the hospital more frequently, the intensity and length of each admission were comparable to those of UC patients. Most admissions in both groups were on the order of one week, reflective of the typical management of moderate-to-severe flares or complications. Between-group summaries of admission counts, intervals between readmissions, and LOS are presented in Table 1.

**Table 1:** Hospitalization frequency and duration in Crohn’s disease vs ulcerative colitis Values are presented as n (%) for categorical variables and mean ± standard deviation or median (range) for continuous variables. Two-sided tests; p<0.05 is considered statistically significant.

Metric	Crohn’s disease	Ulcerative colitis	p-value
Patients, n (% of total 79)	32 (41%)	47 (59%)	-
Total admissions, n (% of 198)	94 (47%)	104 (53%)	-
Median admissions per patient (range)	2 (2-7)	2 (2-5)	-
Mean interval between admissions (days)	172 ± 95	487 ± 134	<0.01
Mean length of stay per admission (days)	8.4 ± 4.1	9.6 ± 5.2	0.18

Reasons for hospitalization

The clinical presentations prompting admission differed somewhat between CD and UC, as shown in Table 2. Abdominal pain was the most common presenting complaint in both groups, reported in over half of admissions for each (52 (55%) of CD admissions; 66 (63%) of UC). Rectal bleeding (hematochezia, reported as rectorrhagia) was also a frequent symptom in both, though it was proportionally more common in CD admissions (35 (37%)) than in UC (27 (26%)). Notably, systemic symptoms such as fever were present in over one-third of Crohn’s admissions, often indicating severe disease or intra-abdominal abscess, whereas high fever was less frequently the primary reason in UC. Instead, profuse diarrhea was a classic hallmark of UC flares, noted in nearly half (48 (46%)) of UC admissions but in a minority of CD cases. Other symptoms such as nausea/vomiting were relatively uncommon primary presentations (observed in a small subset of patients, mostly those with CD and intestinal obstruction or medication side effects).

**Table 2:** Most frequent presentation symptoms by IBD subtype Note: Percentages reflect the proportion of admissions in which the symptom was a leading complaint. Many admissions had multiple concurrent symptoms (e.g., abdominal pain with diarrhea), so categories are not mutually exclusive. CD, Crohn’s disease; IBD, inflammatory bowel disease; UC, ulcerative colitis.

Presentation symptom	CD - admissions with symptoms (% of CD)	UC - admissions with symptoms (% of UC)
Abdominal pain	52 (55%, the most common symptom in CD)	66 (63%, the most common symptom in UC)
Rectal bleeding (hematochezia)	35 (37%)	27 (26%)
Diarrhea	18 (19%, less common in CD flares)	48 (46%)
Fever (≥38°C)	35 (37%)	16 (15%, less common in UC)

In summary, CD flares requiring admission often manifested with acute abdominal pain, sometimes due to partial obstruction or abscess, and/or rectal bleeding, and a significant fraction had febrile presentations suggestive of complicated disease. UC flares, on the other hand, predominantly presented with bloody diarrhea and abdominal cramping pain. These differences align with the underlying pathology: CD can involve transmural inflammation anywhere in the gut (leading to strictures, fistulae, and abscesses, hence pain and fever), while UC is limited to colonic mucosal inflammation (leading to diarrhea and bleeding). Despite these differences, abdominal pain was a common thread in both conditions, highlighting that severe IBD exacerbations frequently cause significant pain.

Departments of admission

We next examined where patients were being admitted for their IBD-related hospitalizations. As expected, the Gastroenterology department managed the majority of cases (overall about 51% of admissions). However, surgical services also played a major role, particularly for CD. Among CD admissions, 35 (37%) were under General Surgery, reflecting the high incidence of complications such as fistulas, abscesses, or obstruction requiring surgical evaluation or intervention. In UC, 31 (30%) admissions were to Surgery, often for severe colitis not responding to medical therapy or for elective colectomy in chronically refractory cases. A smaller proportion of patients in both groups were admitted to Internal Medicine wards (CD 4 (4%), UC 7 (7%)), usually when presenting initially to general medicine or when managed for comorbid conditions alongside the IBD flare. A few patients (<5%) required care in other departments, for example, one CD patient was admitted to Orthopedics for an IBD-related osteoporotic fracture, and one UC patient to a Gynecology ward for severe anemia in pregnancy with colitis flare. These were outliers.

Overall, the data indicate a multidisciplinary approach to IBD hospital care, involving gastroenterologists and colorectal surgeons. Timely surgical consultation for Crohn’s complications was common, whereas UC patients more often remained under medical care unless surgery (colectomy) was needed. Close cooperation between departments is essential given this pattern.

In-hospital complications and outcomes

We recorded key complications that occurred during admissions or were identified as the cause for admission (Table 3). In CD, perianal disease was prominent: perianal abscesses were noted in 6 (18%) CD patients (either as the reason for admission or developing during hospitalization) and perianal fistulas in 4 (4%). Several CD patients required incision and drainage of abscesses or seton placement for fistulas as part of their management. Intra-abdominal abscesses (e.g., ileocecal abscess) were also encountered (in a few cases, managed with antibiotics and percutaneous drainage). Although not formally listed in Table 3, small bowel obstruction due to Crohn’s strictures occurred in a number of cases (10 (10%) CD admissions), these often precipitated urgent surgical admission and were managed either conservatively or with surgery. Three (9%) CD patients were found to have new gastrointestinal neoplasms (two cases of colorectal carcinoma and one small bowel adenocarcinoma) during the study period, discovered either on colonoscopic evaluation during admission or surgery, highlighting the elevated cancer risk in longstanding Crohn’s, although this was not a primary focus of our study.

**Table 3:** Common complications observed during IBD admissions Note: Percentages are patient-based (out of total patients in that subgroup) having the complication at least once during the study period. CD, Crohn’s disease; CRC, colorectal carcinoma; IBD, inflammatory bowel disease; UC, ulcerative colitis.

Complication	CD patients with complications (% of CD)	UC patients with complications (% of UC)
Perianal abscess	6 (18%)	2 (4%)
Perianal fistula	1 (3%)	0 (0%)
Intestinal obstruction/occlusion	3 (9%, small bowel obstruction)	8 (17%, toxic megacolon/colonic dilation)
Severe hemorrhoids	3%	6%
New gastrointestinal neoplasm (detected)	3 (9%, CRC or small bowel cancer)	0 (0%)
*Clostridioides difficile* infection	4 (13%, during ≥1 admission)	5 (11%, during ≥1 admission)
Urgent major surgery required (colectomy or resection)	3 (9%)	6 (13%)

In UC, the most striking complication was acute severe colonic dilation/obstruction (8, 17% of UC patients). This term encompassed instances of toxic megacolon and functional obstruction from severe inflammation. Several UC patients presented with distended abdomen and ceased bowel movements, requiring urgent surgical consultation; fortunately, some improved with medical therapy, while others underwent subtotal colectomy. Another recorded finding in UC was the presence of hemorrhoids (6%), which may reflect symptomatic overlap or comorbid conditions rather than a direct disease complication; this was retained for completeness but is not central to disease-specific morbidity. Perianal abscesses occurred in a small subset of UC patients (2, 4%), illustrating that although classically a Crohn’s complication, severe UC can occasionally involve peri-rectal sepsis (especially in those with longer disease duration or previous therapy that predisposes to infection). UC patients did not develop fistulas except in the perianal region (none had enterocutaneous fistulas, consistent with UC’s mucosal nature). No cases of cytomegalovirus colitis requiring surgery were recorded, and there were no postoperative anastomotic leaks in those who had colectomies.

We also tracked CDI during admissions. Overall, 9 (11% of the cohort) patients had at least one hospitalization complicated by laboratory-confirmed CDI. This included 4 (13%) Crohn’s patients and 5 (11%) UC patients. The rates between CD and UC were not significantly different. CDI often prolonged the hospital stay and necessitated infection control measures and vancomycin or fidaxomicin therapy. One patient with UC had a particularly severe episode of fulminant colitis compounded by CDI, requiring urgent colectomy. This concurs with literature noting that UC patients are especially prone to CDI during flares [4]. Fortunately, there were no deaths in our series; all patients were discharged alive, and the 30-day post-discharge mortality was zero. However, the morbidity from complications was considerable, with about 15% of patients eventually needing surgery (colectomy in UC or resection in CD).

In summary, CD admissions were often complicated by penetrating disease (abscesses and fistulae), whereas UC admissions were more likely to involve fulminant colitis with risk of toxic megacolon. Both diseases occasionally faced overlapping issues such as perianal infections and the opportunistic infection caused by *C. difficile*. A notable proportion of patients in both groups eventually required surgical intervention, reflecting that readmissions often signaled aggressive disease unresponsive to medical therapy.

## Discussion

Frequent hospital readmissions in IBD pose significant challenges in patient management and healthcare utilization. This single-center study provides a detailed comparison of CD and UC with regard to readmission patterns, reasons for hospitalization, and complications. Our findings confirm some expected differences between CD and UC while also highlighting areas of common concern. In this context, it is valuable to compare our results with the wider literature and discuss implications for clinical practice.

Readmission frequency in CD vs UC

We observed that CD patients were readmitted much sooner and more often than UC patients. This aligns with prior research suggesting that CD can be more refractory to standard treatments and prone to relapse. Other studies have similarly found a modestly higher readmission risk for CD compared to UC, even after adjusting for other factors [5]. One reason is the transmural nature of CD, which can lead to complications (abscesses and strictures) that necessitate repeat hospital care. In contrast, UC, when medically controlled or cured by colectomy, may result in longer remission periods without hospitalization [6]. Interestingly, despite more frequent admissions, the length of each hospital stay did not differ between CD and UC in our study. This suggests that once hospitalized, UC flares can be just as severe and require comparable treatment duration (e.g., intravenous steroids or biologics, and work-up for surgery) as Crohn’s flares. Thus, while preventing the occurrence of relapses is especially crucial in CD, aggressive management of an acute severe episode is equally important in UC.

Presenting symptoms and clinical course

The symptom profiles we documented mirror classical teaching: UC’s hallmark is diarrhea with bleeding, whereas Crohn’s often presents with pain and less overt bleeding. Nonetheless, a sizeable fraction of Crohn’s admissions in our series had rectorrhagia (37%), indicating colonic involvement or severe perianal disease. Conversely, abdominal pain was nearly ubiquitous in UC flares as well, reminding clinicians that pain can be a feature of UC, especially if accompanied by colonic distension or IBS-like sensitivity. Patients admitted for pain control may be at a higher risk for readmission compared to those admitted for other complications [7]. Fever tended to be a marker of complicated Crohn’s (e.g., abscess) in our patients. This is corroborated by other studies that identified fever on admission as a predictor of IBD complications and the need for surgery. The overlap in symptoms means that thorough evaluation (including imaging for abscess or megacolon, and stool studies for infection) is warranted for any severe IBD flare presenting to the hospital.

Risk factors for readmission

A key aim of our study was not just to describe what happened during these admissions but to contextualize why some patients end up in a cycle of recurrent hospital care. Our results must be interpreted with an understanding that certain risk factors predispose IBD patients to readmission. Consistent with the literature, we highlight three modifiable factors: psychiatric comorbidities, corticosteroid use, and CDI. 

Mental health support is another essential component of multidisciplinary care. Research by Byrne et al. indicates that patients with IBD often experience anxiety and depression, which can severely affect their treatment outcomes and hospital utilization [8]. Though we did not formally measure depression or anxiety in our retrospective data, numerous studies have underscored their impact. For instance, a 2017 analysis of the Nationwide Readmissions Database found that anxiety disorders and depression increased 90-day readmission odds by ~30% in both CD and UC [1]. Psychological stress can worsen IBD symptoms and affect medication adherence. Additionally, patients with depression or anxiety might have poorer self-care and lower engagement in follow-up, as suggested by higher emergency department use. A systematic review has reported that psychological comorbidities such as depression significantly enhance the risk for readmission. The probability of readmission increases as psychological distress negatively affects patients' disease management after discharge [9]. Integrating psychological support into the standard care model has been recommended, as psychological distress can exacerbate the physical symptoms of IBD [10]. Screening and treating psychiatric illness could reduce readmissions. Our anecdotal observation was that several frequently readmitted patients had underlying depression or high stress levels, which likely contributed to difficult-to-manage IBD.

Corticosteroids remain a mainstay for acute IBD flares and can induce rapid symptomatic improvement, which may temporarily reduce short-term hospital utilization. However, their repeated or prolonged use reflects persistently active disease and is associated with an increased risk of complications and future readmissions. Chronic steroid use was associated with a markedly higher 90-day readmission risk in the Middle East cohort [3]. Steroids also predispose patients to infections (including CDI) and other adverse effects that can prompt hospital care [4]. Therefore, while appropriate for acute flares, steroids should be tapered as soon as possible and replaced with steroid-sparing maintenance therapies (immunomodulators or biologics). Our data showed that many UC patients were admitted with steroid-refractory flares, and several Crohn’s patients had complications (such as osteoporosis fractures or diabetes) likely related to past steroid use. Optimizing outpatient therapy to minimize steroid exposure (e.g., using biologic therapy earlier) may help break the cycle of readmissions.

CDI in the context of IBD flares is a known trigger for severe disease and can be difficult to distinguish from an uninfected flare. We found 9 (11%) patients who had CDI during an admission, consistent with other reports that 5%-10% of IBD hospitalizations involve CDI. [2]. CDI not only prolongs the index hospital stay but may also precipitate rapid readmission if not fully eradicated or if IBD therapy is held due to infection. Feuerstein et al. reported that UC patients undergoing surgery with active CDI had higher readmission rates, reflecting the additive morbidity [11]. Therefore, if an IBD patient tests positive for CDI, prompt initiation of oral vancomycin therapy is recommended in line with current guidelines. Furthermore, infection control and judicious antibiotic use are key prevention strategies. In our hospital, we implemented a protocol to test all patients with IBD flare diarrhea for CDI and found it helpful. Furthermore, infection control and judicious antibiotic use are key prevention strategies.

Beyond these factors, other considerations include socioeconomic and healthcare system factors. Our study being single-center in Romania, most patients were local and insured; however, some lived far and may have had limited access to specialist follow-up, potentially increasing readmission risk. Studies in the US have found that younger patients and those of lower income or on Medicare have higher readmission rates [2], possibly due to disparities in outpatient care continuity. While our dataset did not capture income, it is plausible that enhanced post-discharge support (such as IBD nurse telephone follow-ups or timely clinic visits) could reduce bounce-backs. Indeed, failure to attend Gastroenterology follow-up after discharge has been associated with early readmission in IBD. We attempt to facilitate early post-hospital appointments for our patients; still, a structured “transition of care” program for IBD is an area for improvement. The absence of timely follow-up significantly increases the risk of readmission, as noted in quality improvement efforts across clinical frameworks [12,13]. Implementing care strategies that include targeted nutritional assessments and mental health support, especially for the aging IBD population, can effectively address these risks [14].

Clinical implications

For clinicians, our findings reinforce that CD patients require vigilant monitoring after discharge given their high relapse tendency. Early intervention on symptoms before they escalate could avert some admissions. In UC, identifying patients at risk of severe flares (e.g., those with previous hospitalizations, extensive colitis, or prior steroid use) is important; these patients might benefit from expedited escalation to biologic therapy or even elective surgery before emergency surgery becomes necessary. Our data also underscore the importance of a multidisciplinary approach: gastroenterologists, surgeons, stoma nurses, dietitians, mental health professionals, and IBD nurses all have roles in managing complex IBD to prevent readmissions. For example, an IBD case manager could ensure patients understand medication changes, have access to therapy (biologic infusion appointments, etc.), and know to seek early outpatient care if symptoms recur, rather than waiting until hospital-level severity. De Dycker et al. emphasize the importance of multidisciplinary care pathways, particularly in scenarios such as pregnancy, where patients may experience exacerbations of their conditions [15]. Surgical intervention adds complexity to the management of IBD, necessitating close integration between medical and surgical teams. Louis et al. highlight that structured collaborations in IBD units can help determine optimal timing for surgeries and minimize postoperative complications [16]. Additionally, several studies indicate that multidisciplinary planning improves surgical outcomes, particularly in patients requiring stoma creation due to severe IBD [17,18]. 

Comparison with other studies

The patterns we observed are generally congruent with other published cohorts. Micic et al. [2] found 30-day readmission in 7% of IBD discharges and noted that younger age and Crohn’s diagnosis were associated with readmission, akin to our observation of frequent Crohn’s relapses in a relatively young cohort (mean age: ~40 years). They also reported that performing surgery during the index admission was associated with lower readmission risk, presumably by resolving the issue (we similarly saw that some of our post-colectomy UC patients had no further admissions). A study mentioned that veteran patients often experience a median time of 26 days to re-hospitalization, indicating that a significant number are readmitted early in the post-discharge period, often linked to complications such as infections and exacerbations of disease [19]. Mudireddy et al. [20] distinguished between early (30-day) and late readmissions in IBD, finding that disease activity and complications drive early readmissions, while medication non-adherence plays a role in later ones. This highlights that once immediate issues are dealt with, long-term adherence and psychosocial support become critical - something for us to address in our setting as well.

Limitations

We acknowledge several limitations in our study. Firstly, the sample size is modest (79 patients), reflecting a single-center experience. Thus, our power to detect certain associations (e.g., the effect of specific medications on readmission) was limited, and our percentages for complications must be interpreted with caution in light of the small denominators. Secondly, the retrospective design relies on the accuracy of recorded data; it is possible that some readmissions to other hospitals were not captured, and symptom documentation may have varied. The modest sample size and single-center nature of the study limit statistical power and external validity. Certain findings, such as the higher rate of rectal bleeding in CD, should therefore be interpreted cautiously and considered hypothesis-generating rather than definitive. However, as ours is the main referral hospital in the region for IBD, we believe a few events were missed. Thirdly, we did not formally collect data on some known risk factors such as smoking status, specific outpatient therapies, or mental health diagnoses due to inconsistent documentation. These factors could confound readmission risk (for example, smoking is known to exacerbate CD [21]), and depression correlates with higher healthcare utilization. Prospective studies would be ideal to gather those patient-level details. Fourthly, because we focused on readmissions over 5 years, our analysis did not specifically differentiate 30-day vs 90-day readmissions in detail. All multiple admissions were considered, which is a slightly different metric than standardized readmission rates; thus, our findings complement but do not directly mirror studies using fixed time windows. Finally, being a single-center study, our results may not generalize to all settings; for instance, local practice patterns (threshold for admission, availability of biologics, and patient demographics) can influence readmission rates. Nonetheless, the consistency of our core findings with larger studies lends credibility to their broader applicability.

Although many of the risk factors associated with IBD readmission, such as corticosteroid use, psychiatric comorbidities, and CDI, have been previously described in multicenter and nationwide studies, our work contributes unique regional data from Eastern Europe. This perspective is important because access to biologic and small-molecule therapies, healthcare infrastructure, and follow-up systems differ substantially from those reported in Western cohorts. By providing detailed admission-level information and focusing on recurrent admissions within a real-world Romanian tertiary center, this study complements existing literature and helps contextualize global IBD readmission trends.

Beyond sample size and design constraints, our study did not include systematic data on outpatient care quality, adherence, or psychosocial status, which are recognized contributors to hospital readmission. These aspects could not be retrospectively quantified from chart review but represent important targets for future prospective research. Similarly, details on biologic and small-molecule therapies were incompletely captured, reflecting the transitional nature of treatment availability during the study period. Future multicenter studies integrating these parameters would enable a more comprehensive assessment of the multifactorial drivers of IBD readmission.

## Conclusions

Hospital readmissions are common in IBD and show distinct patterns by subtype: over five years, CD had more frequent and earlier readmissions than UC. Admissions were mainly for abdominal pain, gastrointestinal bleeding, and diarrhea, with complications such as perianal abscesses in CD and toxic megacolon in UC contributing to morbidity.

Preventable readmissions may be reduced by optimizing maintenance therapy (minimizing steroid exposure), ensuring timely post-discharge follow-up, screening and treating *C. difficile*, and addressing psychiatric comorbidity within a coordinated, multidisciplinary pathway that also may permit timely surgery when indicated. These targeted strategies aim to keep patients in remission, improve quality of life, and lessen healthcare burden; future prospective, multicenter studies should test their effectiveness.
